# Every Coin Has Two Sides: Reactive Oxygen Species during Rice–*Magnaporthe oryzae* Interaction

**DOI:** 10.3390/ijms20051191

**Published:** 2019-03-08

**Authors:** Yanjun Kou, Jiehua Qiu, Zeng Tao

**Affiliations:** 1 State Key Lab of Rice Biology, China National Rice Research Institute, Hangzhou 311400, China; qiujiehua@caas.cn; 2 College of Agriculture and Biotechnology, Zhejiang University, Hangzhou 310058, China

**Keywords:** reactive oxygen species (ROS), *Magnaporthe oryzae*, rice blast, disease resistance, rice-*Magnaporthe oryzae* interaction, NADPH oxidase OsRbohB, OsMT2b, NADP-malic enzyme2 (Os-NADP-ME2)

## Abstract

Reactive oxygen species (ROS) are involved in many important processes, including the growth, development, and responses to the environments, in rice (*Oryza sativa*) and *Magnaporthe oryzae*. Although ROS are known to be critical components in rice–*M. oryzae* interactions, their regulations and pathways have not yet been completely revealed. Recent studies have provided fascinating insights into the intricate physiological redox balance in rice–*M. oryzae* interactions. In *M. oryzae*, ROS accumulation is required for the appressorium formation and penetration. However, once inside the rice cells, *M. oryzae* must scavenge the host-derived ROS to spread invasive hyphae. On the other side, ROS play key roles in rice against *M. oryzae*. It has been known that, upon perception of *M. oryzae*, rice plants modulate their activities of ROS generating and scavenging enzymes, mainly on NADPH oxidase OsRbohB, by different signaling pathways to accumulate ROS against rice blast. By contrast, the *M. oryzae* virulent strains are capable of suppressing ROS accumulation and attenuating rice blast resistance by the secretion of effectors, such as AvrPii and AvrPiz-t. These results suggest that ROS generation and scavenging of ROS are tightly controlled by different pathways in both *M. oryzae* and rice during rice blast. In this review, the most recent advances in the understanding of the regulatory mechanisms of ROS accumulation and signaling during rice–*M. oryzae* interaction are summarized.

## 1. Introduction

ROS are highly reactive reduced forms of oxygen molecules, including superoxide (O^2−^), hydrogen peroxide (H_2_O_2_), hydroxyl radical (OH^−^), and singlet oxygen (O_2_) [1,2,3,4,5,6]. ROS are produced dependent on several classes of enzymes, including NADPH (nicotinamide adenine dinucleotide phosphate) oxidases, peroxidases, and oxidases, and cell compartments, which are mainly dominated by chloroplasts, mitochondria, and peroxisomes. The ROS generation by the enzymes and compartments has been summarized in some excellent recent reviews [6,7,8,9]. Among the ROS-generating enzymes, NADPH oxidases were best characterized in both *Magnaporthe oryzae* and rice. The NADPH oxidases (Nox1-Nox4, Nox5, and dual oxidase Duox) have FAD and NADPH-binding sites and an oxidase domain responsible for O_2_^−^ generation [10]. In *M. oryzae*, there are three putative NADPH oxidases: Nox1, Nox2, and Nox3 [11]. Both Nox1 and Nox2 are known for ROS production in *M. oryzae* [11]. In rice, nine NADPH oxidases have been annotated. Among these NADPH oxidases, OsRbohB (a homolog of the catalytic subunit pg91^phox^) is well known as an important component in disease resistance against rice blast [10,12,13,14].

Rice blast is caused by the filamentous ascomycete, *M. oryzae*. It is one of the most serious diseases of crops and the top 10 fungal diseases in plants [15]. To establish successful infections in rice plants, *M. oryzae* has evolved highly sophisticated and specific infection strategies. Generally, *M. oryzae* utilizes highly specialized appressoria, which are produced from three-celled conidia on the rice surface, to gain entry therein [16,17]. The appressorium formation in *M. oryzae* requires temporal-spatial ROS production in both the germ tube tip and immature appressorium integrated with other signals. In addition, ROS production is involved in the maintenance of the F-actin network, as well as septin-dependent assemblies of the exocyst at the appressorial pore to facilitate the penetration. Once inside the rice cells, *M. oryzae* differentiates into invasive hyphae and then spreads to neighboring cells, resulting in the formation of lesions. During invasive growth, *M. oryzae* needs to elude the plant immunity, including suppressing ROS accumulation, to adapt to the host milieu [6,14,18]. On the other hand, the rice plants equip variable and remarkable defense systems to resist the potential attacks of *M. oryzae*. One general priming and critical event in rice immunity is the production of ROS to resist the infections of *M. oryzae* [2,6,14]. In the current review, the recent progresses in ROS production and signaling in both *M. oryzae* and rice during *M. oryzae*–rice interaction are summarized.

## 2. ROS Play Dual Roles in the Pathogenesis of *M. oryzae*

### 2.1. ROS Accumulations are Required for Infection Structure Formation and Penetration in M. oryzae

**Figure 1:**
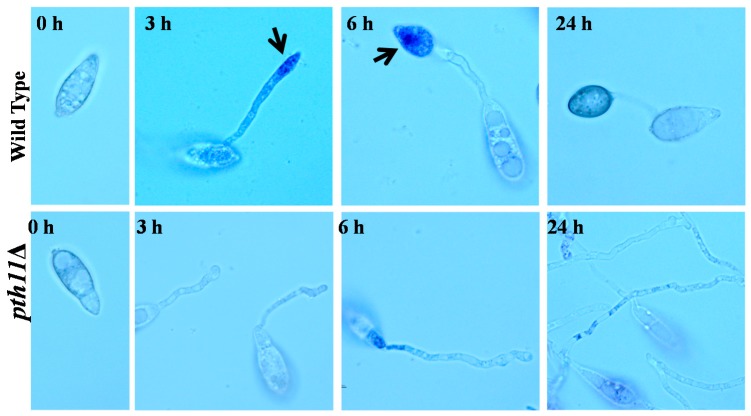
ROS accumulate in the tip of the germ tube and immature appressorium. Nitroblue tetrazolium (NBT) staining was performed in wild type or *pth11*Δ strains during the appressorium formation [20]. Arrows highlight the ROS in the tips of its germ tubes and immature appressoria. Bar = 5 μm.

ROS are produced in the metabolic processes of *M. oryzae* and act as signals in a variety of developmental pathways, including the formation of infection structures. *M. oryzae* forms a specialized infection structure called the appressorium, which ruptures the host cuticles with high turgor, and drives a penetration peg into the underlying host cell. During appressoria morphogenesis, *M. oryzae* accumulates high levels of endogenous ROS in the tips of its germ tubes and immature appressoria (Figure 1) [11,19,20]. The scavenging of the ROS delays the differentiation of the appressoria, and also changes the morphology of the appressoria in *M. oryzae*. Recently, our studies reveal that the ROS accumulation is irregular and disrupted in the deletion mutants of *PTH11* (Figure 1) and its downstream signaling components, *MAGB* and *PMK1* [20,21]. Pth11 is an important G-protein coupled receptor required for the appressorium formation in *M. oryzae* [20,22]. Moreover, treatments with antioxidants induce functional appressoria differentiation in the *pth11*Δ strain [20], suggesting that the altered ROS accumulation is a probable cause of appressorium formation defect in *pth11*Δ.

**Figure 2:**
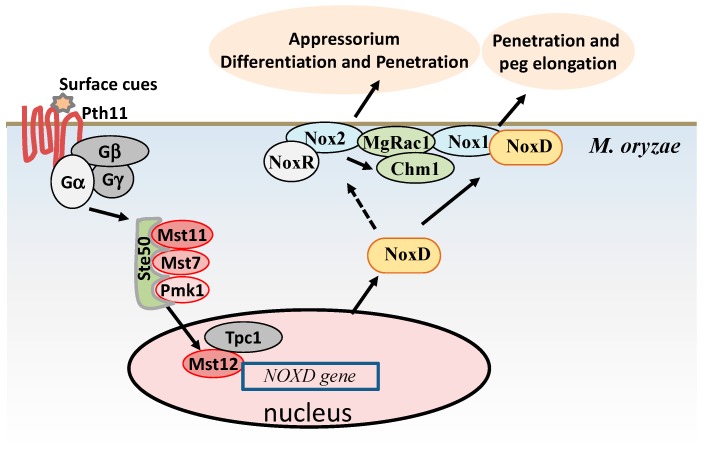
Model of NADPH oxidases-mediated appressorium formation and penetration in *M. oryzae*. The host surface cues are sensed by the G-protein coupled receptor, Pth11. After recognition of the cues, the Gα subunit binds to GTP (guanosine triphosphate) and dissociates from the Gβγ dimer and the induction of downstream MAPK (mitogen-activated protein kinase) cascades signaling occurs [21]. A component of the MAPK cascades signaling pathway, Mst12, interacts with the Zn(II)_2_Cys_6_ transcriptional regulator, Tpc1, to regulate the expression of the orthologue of the *P22^phox^* subunit (*NOXD*) of the NADPH oxidase complex. NoxD interacts with Nox1 and may be with Nox2 indirectly to control the elongation of the penetration peg. Both Nox1 and Nox2 interact with small GTPase, MgRac1, and may be regulated by MgRac1. In addition, the synthesis of ROS by the NoxR-Nox2 NADPH oxidase complex is required for the maintenance of the F-actin network and septin-dependent assembly of the exocyst at the appressorium pore to initiate plant infection.

It is well known that gp91^phox^ (Nox2) becomes active after the assembly of four cytosolic regulatory components (p47^phox^, p67^phox^, p40^phox^, and Rac2) with the integral membrane proteins cytochrome b_558_ (composed of catalytic subunit gp91^phox^ and p22^phox^) in humans [23]. In *M. oryzae*, three putative homologs of the catalytic subunit gp91^phox^, Nox1, Nox2, and Nox3, have been characterized [11]. Nox1 is required for the ROS accumulations during appressorium morphogenesis and the elongation of the penetration peg [11,24]. Nox2 is necessary for the ROS accumulations in the hyphal tip and immature appressorium to form functional appressorium and the development of the penetration peg [11,24,25]. In *M. oryzae*, the successful infection of plants requires the maintenance of the F-actin network and septin-dependent assembly of the exocyst through the septin GTPases (sep3, Sep4, Sep5, and Sep6) at the appressorial pore. These processes require the synthesis of ROS by the Nox2-NoxR (p67^phox^ homolog) complex to regulate the proper localization of Sep6 and organization of the exocyst complex [24,26]. Notably, Nox2 is known to regulate the localization of Chm1 kinase to possibly activate the PMK1 MAP (Mitogen-activated protein kinase) kinase pathway during the appressorium formation and penetration [26,27]. Similar to rice, the activities of both Nox1 and Nox2 may be regulated by small GTPase, MgRac1 [25]. The activity of MgRac1 is positively related to ROS accumulation in the hyphal tips [25]. Furthermore, MgRac1 directly interacts with Nox1, Nox2, and Chm1 to regulate ROS accumulation and the appressorium formation in *M. oryzae* [25]. In addition, the p22^phox^ homolog, NoxD, interacts with Nox1 to play roles in the cytoskeletal re-modeling in the appressorium [28]. The expression of *NOXD* is directly regulated by the Tpc1 (transcription factor for polarity control 1) via an interaction with transcriptional factor Mst12 [28]. Therefore, the Tpc1 is required for NADPH oxidase dependent appressorium re-polarization through the control of the spatial and temporal regulation of the cortical F-actin during penetration [28]. All of the aforementioned findings reveal that *M. oryzae* needs an intricate physiological redox balance to regulate the septin assembly and formation of the F-actin network, which leads to functional appressorium formation and penetration (Figure 2). 

### 2.2. Neutralization of the Host-Derived ROS is Required for Invasive Growth in Virulent Rice–M. Oryzae Interaction

As previously mentioned, ROS accumulation is required for functional appressorium formation and penetration in *M. oryzae*. However, once inside the rice cells, *M. oryzae* requires effective anti-oxidant defense systems to scavenge or detoxify the host-derived ROS to spread invasive hyphae by making a conclusion of the phenotypes of many mutants. In *M. oryzae*, secreted ROS scavenging enzymes, such as peroxidase and laccase, may contribute to the pathogenicity. The deletion mutants of a fungal-specific protein gene *DES1*, transcription factor *MoAP1*, oxidoreductase enzyme gene *MoTRX2* (a target of the *MoAP1*), and protein phosphatase gene *MoYVH1*, exhibit stronger ROS accumulation, induction of defense response genes, and reduced pathogenicity [29,30,31,32]. It is believed that the ROS accumulations in the *des1*Δ, *Moap1*Δ, *Motrx2*Δ, and *Moyvh1*Δ mutants during *M. oryzae* infection may be related to lower production or activation levels of extracellular peroxidase and laccases [29,30,31,32]. Similarly, the deletion mutant of the soluble NSF (*N*-ethyl-maleimide-sensitive protein) attachment protein receptor, *MoVAM7*, which is required for the secretory transport of laccases, affects the ROS accumulation in the hypha and the pathogenicity [33]. Moreover, the mutants of ROS detoxifying enzymes encoding genes in *M. oryzae*, such as glutathione peroxidase domain-containing gene *HRY1*, glutathione reductase gene *GTR1*, and nitronate monooxygenases gene *NMO2*, fail to inhibit host ROS accumulation during infection [18,34,35]. Although these three enzymes likely exhibit ROS detoxifying activities, questions regarding how the fungal resident enzymes regulate the ROS accumulations in the host remain to be answered. A similar question can be raised about how the NAD (nicotinamide adenine dinucleotide)-dependent histone deacetylase MoSir2 regulates the expression of a fungal mitochondrion localized enzyme to suppress defense and the ROS accumulation in the host [36]. 

Moreover, there are an abundance of mutants found in *M. oryzae*, which fail to suppress the ROS accumulation in the host without having obvious relationships with the ROS generating or scavenging components [37,38,39,40,41,42,43,44]. Collectively, the results of previous studies have strongly implied that failures in ROS detoxification are highly likely to cause virulent defects in *M. oryzae*. However, this hypothesis is not supported by the observations of ROS levels using Grx1-roGFP2 in *M. oryzae* [19,45]. In regard to this issue, Samalova et al. proposed that *M. oryzae* has the ability to tolerate extreme oxidative stress, and does not undergo oxidative stress in *planta* [19]. One explanation may be that the function of these genes is more than just ROS detoxification in the ROS signaling of the host. Further elucidations of these mutants will provide important clues to understand the ROS generating and signaling in the virulent rice–*M. oryzae* interaction. 

## 3. ROS as Key Players against *M. oryzae* Attacks in Rice

### 3.1. OsRbohB as an Important Component against Rice Blast

ROS play important roles in both the first line of defense termed as pathogen-associated molecular patterns (PAMPs) triggered immunity (PTI) and the second line of defense related to effector-triggered immunity (ETI) [2,6,9,14,46,47,48]. By the perception of the PAMPs, pattern recognition receptors (PRRs) activate a variety of immune responses, including the rapid and strong production of ROS through OsRbohB and other ROS generating components, to trigger PTI in rice [49,50]. In the second line of defense, plant intracellular immune receptors directly or indirectly recognize the specific pathogen effectors to induce ETI. In this situation, rice plants activate a series of signaling pathways, which lead to hypersensitive responses (HR). The HR activation involves the ROS burst, callose deposition, induction of the expression of pathogenesis-related protein genes, and programmed cell death [14,49]. In rice, OsRbohB is required for ROS burst in the HR during immunity against rice blast [12,13,51,52]. In addition, OsRbohB is also involved in the ROS generations, which are regulated by lesion mimic genes and plant hormones to contribute to the resistance of rice against *M. oryzae* [53,54].

#### 3.1.1. Small GTPase OsRac1 Plays Dual Roles in the Induction of OsRbohB-Dependent ROS and the Suppression of ROS Scavenging during Immunity against Rice Blast

**Figure 3:**
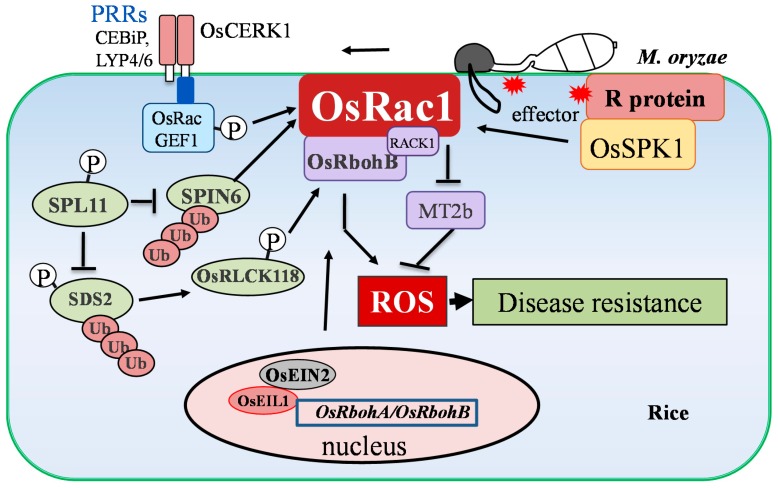
OsRbohB as an important component against rice blast. Small GTPase, OsRac1, can be activated by both pathogen-associated molecular patterns (PAMPs) triggered immunity (PTI) and effector-triggered immunity (ETI) to induce OsRbohB-dependent ROS production and suppress ROS scavenging. By sensing the PAMPs or effectors, the OsRac1 is activated to induce ROS accumulation by NADPH oxidase OsRbohB activation and suppress ROS scavenging by down regulating the expression of *OsMT2b*. In addition, Spl11 (Spotted leaf 11) regulates the activity of OsRbohB through SDS2 (SPL11 cell-death suppressor) and OsRLCK118 to suppress ROS production. Moreover, the ethylene signaling components, OsEIN2, and its downstream transcription factor, OsEIL1, could regulate the expression of *OsRbohA*/*OsRbohB* to regulate ROS generation during rice–*M. oryzae* interaction.

Some of the PAMPs from *M. oryzae* have been known to induce ROS in rice [55,56]. Chitin derived from fungi is one of the best-characterized PAMPs [56]. The perception of chitin in rice depends on a ligand induced complex between the LysM-containing proteins, CEBiP (chitin elicitor-binding protein) or LYP4/6 and OsCERK1 [57,58,59,60,61,62]. Upon the perception of chitin, the OsCERK1 forms a complex with OsRacGEF1/2 at the endoplasmic reticulum. Then, the complex is transported to the plasma membrane, where it interacts with the OsRac1 to activate a series of responses, including ROS production and expression of defense genes [63,64]. Recently, a new report suggests that the chitinase, MoChai, binds chitin to suppress the chitin-triggered plant immune response and ROS generation. Conversely, MoChai also acts as PMAP to induce PTI [48,65]. It is unknown whether the MoChai is involved in the OsRac1-mediated signaling. In addition, OsRac1 may directly interacts with several disease resistance (R) proteins (including Pit, Pib, Pi9, and Pita), which recognize the effectors from the *M. oryzae* and trigger ETI [51,66,67,68]. It has been noted that inductions of the HR and ROS burst by Pit are dependent on the OsRac1 [66,67]. Furthermore, recent studies showed that Pit or Pia activates OsRac1 by GEF OsSPK1 (a DOCK family guanine nucleotide exchange factor) on the plasma membrane [68]. All of the aforementioned results demonstrate that OsRac1 can be activated by both PTI and ETI to induce ROS accumulations during rice blast (Figure 3).

It has been shown that OsRac1 regulates ROS production through its interaction with NADPH oxidase, OsRbohB [10,12,13,52]. Constitutively active OsRac1 induces ROS accumulation by regulating OsRbohB activity and enhances the resistance of rice to *M. oryzae* [10,12,51,69]. Meanwhile, the OsRac1-interacted protein, RACK1A, which plays a role in the resistance against rice blast infection, is also known to interact with OsRbohB [52]. The *OsRbohB*-knockdown rice plants increase the susceptibility to virulent *M. oryzae* isolates, suggesting that OsRbohB is essential for basal resistance [13]. However, it remains to be seen whether knockdown *OsRbohB* compromises the *R* gene mediated disease resistance. In addition to inducing ROS burst, OsRac1 down regulates the *metallothionein* gene (*OsMT2b*) to suppress ROS scavenging [70]. The OsMT2b has superoxide- and hydroxyl radical-scavenging activities and functions as a negative regulator of rice blast resistance [70]. Taken together, these findings provide fascinating evidence that OsRac1 plays dual roles in the induction of ROS production and the suppression of ROS scavenging (Figure 3).

#### 3.1.2. Rice Blast Resistance Pathways Involved in *spotted leaf 11* (*spl11*) could be Integrated with ROS Signaling via OsRbohB

ROS generation must be tightly controlled to avoid detrimental effects on rice plants. It must be produced in appropriate amounts, with the correct localization, and at the right time. Abnormal accumulation of ROS may cause defects in the rice, such as lesion-mimics and spots. Over the last two decades, many lesion-mimic mutants in rice have been identified with an abnormal accumulation of ROS and programmed cell death [71,72,73,74,75,76,77,78,79,80,81,82,83,84]. The majority of these lesion-mimic mutants have displayed enhanced disease resistance, including resistance against rice blast [72,74,76,77,78,79,80,83]. However, spontaneous lesions in rice plants with increased ROS accumulation and susceptibility to *M. oryzae* infection have been observed, suggesting that highly accumulated ROS in rice may not directly kill *M. oryzae* during infection [85]. 

*SPL11* encodes an E3 ubiquitin ligase [86], which is one of best characterized lesion-mimic mutant genes found in rice. It has been determined that the *spl11* mutant accumulates increased H_2_O_2_ and confers resistance to rice blast [73,87]. Spl11 interacts with a Rho GTPase activating protein, SPIN6, to ubiquitinate and degrade SPIN6 through the 26S proteasome pathway [53]. RNAi silencing or knockout of the *SPIN6* gene elevates the chitin- and flg22-mediated defense responses and the resistance to rice blast [53]. Moreover, SPIN6 functions as a GTPase-activating protein (GAP) towards the OsRac1-associated defense response [53]. Recently, it has been found that Spl11 regulates the activity of the OsRbohB through the SDS2 and OsRLCK118 [88]. *SDS2*, encoding S-domain receptor-like kinase, is an *Spl11* cell-death suppressor, as well as a positive regulator of resistance to rice blast [88,89]. SDS2 interacts with and phosphorylates OsRLCK118, which positively regulate rice blast resistance by phosphorylating the OsRbohB to stimulate the generation of ROS [88]. These results highlight that the pathways involved in the *spl11* mutant could be integrated with the ROS signaling via the OsRbohB (Figure 3). In addition, detailed analyses between ROS production and other lesion-mimic mutant genes would be expected.

#### 3.1.3 Plant Hormones May Regulate ROS Accumulation through OsRbohA/OsRbohB during Rice-*M. oryzae* Interaction

Other possible regulators of ROS generation (for example, plant hormones) during resistance against rice blast have also been investigated during recent years. Plant hormones, such as salicylic acids (SA), jasmonates (JAs), and ethylene (ET), are known as signals of diverse array of defense responses in rice [4,90]. Treating rice plants with SA or methyl-jasmonate (MeJA) induces ROS accumulation and enhances the resistance against rice blast [91,92]. The SA-deficient NahG rice plants, which have greatly reduced SA due to the overexpression of the bacterial *nahG* gene, increase the levels of superoxide and H_2_O_2_ and the susceptibility to the virulent isolate of *M. oryzae* [85]. Also, it has been shown that the ethylene (ET) biosynthesis is important for rice plants’ resistance to rice blast [93,94]. Recently, the ethylene signaling components, OsEIN2, and its downstream transcription factor, OsEIL1, have been determined to positively regulate ROS accumulation and disease resistance to rice blast [54]. Interestingly, the OsEIL1 directly binds to the promoters of *OsRbohA*/*OsRbohB* [54], suggesting that plant hormones may possibly regulate ROS accumulation through OsRbohA/OsRbohB during rice–*M. oryzae* interaction (Figure 3).

### 3.2. NADP-Malic Enzyme 2 Regulates ROS Production during M. oryzae Infection

**Figure 4:**
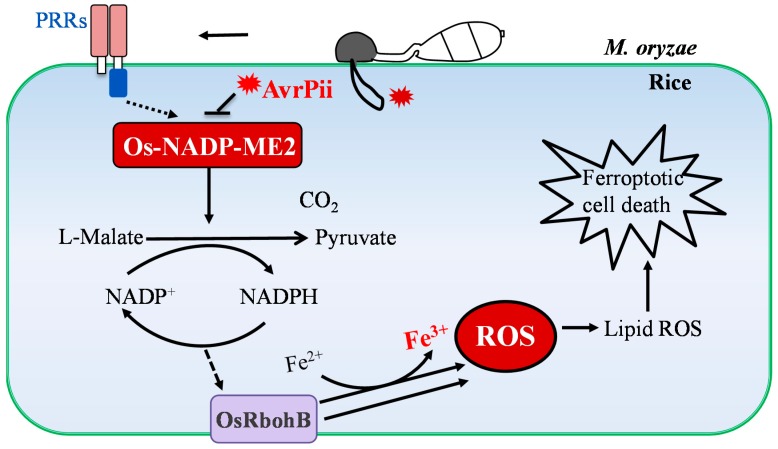
NADP-malic enzyme 2 regulates ROS production during *M. oryzae* infection. Os-NADP-ME2 is involved in the AvrPii-triggered ROS inhibition, chitin-triggered ROS burst, and ROS-dependent ferroptotic cell death. The malic enzyme activity of Os-NADP-ME2 is activated by chitin-treatment, while it can be suppressed by AvrPii. The activated Os-NADP-ME2 catalyzes malic acid into pyruvic acid, which generates NADPH, and subsequently produces ROS to induce the ROS-dependent ferroptotic cell death in rice.

In general, evidence suggests that avirulent pathogens induce a strong ROS burst and HR cell death [2,14]. The most recent study has highlighted that the accumulation of ferric ions and ROS-dependent ferroptotic cell death occur during avirulent *M. oryzae* infection [95]. Suppressing iron-dependent ROS accumulation and lipid peroxidation with ferroptosis inhibitors and the NADPH oxidase inhibitor abolish the HR cell death during avirulent *M. oryzae* infection [95]. In contrast, the HR cell death is induced in virulent *M. oryzae* infection by erastin, which can trigger iron-dependent ROS burst, the accumulation of ferric ions, and glutathione depletion [95]. Notably, chitin fails to induce the accumulation of ferric ions. The accumulation of ROS and ferric ions in the HR cell death is dependent on the NADP-malic enzyme 2 (Os-NADP-ME2) (Figure 4) [95,96]. Knock out of the *Os-NADP-ME2* gene in a resistant rice line disrupts the immunity against *M. oryzae*, suggesting that *Os-NADP-ME2* plays an important role in the exclusion of pathogens. Furthermore, recent research has shown that Os-NADP-ME2 is involved in AvrPii-triggered ROS inhibition [96]. The interaction of Os-NADP-ME2 and AvrPii suppresses the malic enzyme activity of Os-NADP-ME2 and NADPH production to attenuate the host ROS burst in virulent *M. oryzae* infection [96]. As indicated above, the Os-NADP-ME2 may directly regulate the ROS production during immunity. It would be interesting to determine the virulence functions of the AvrPii, as well as the functions of the Os-NADP-ME2 in Pii-mediated disease resistance. Additionally, Os-NADP-ME2 is also required for chitin-triggered ROS burst [95,96]. A detailed correlation between the PAMPs-induced ROS production and Os-NADP-ME2 activity would be expected. Furthermore, it is worthwhile to clarify whether the Os-NADP-ME2 regulates ROS production during immunity through OsRbohB.

### 3.3. Other ROS Accumulation Mechanisms during Rice–M. Oryzae Interaction

**Figure 5:**
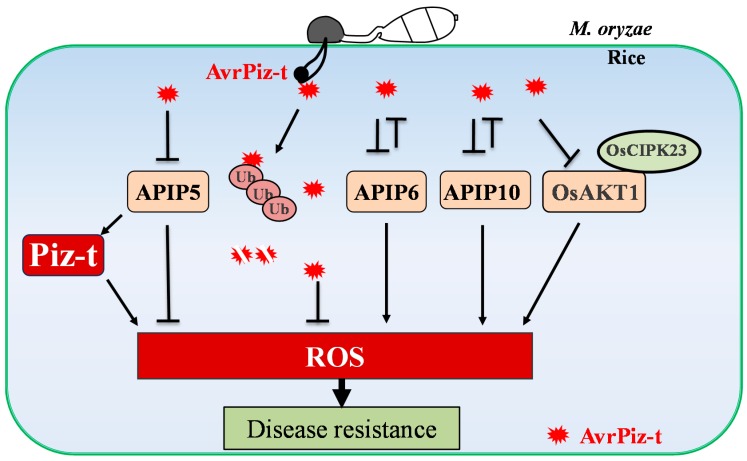
AvrPiz-t and AvrPiz-t targeted proteins regulate ROS accumulation during immunity. APIP5, APIP6, and APIP10 are AvrPiz-t interacting proteins. OsAKT1, which interacts with AvrPiz-t, is a rice plasma-membrane-localized K^+^ channel protein. Silencing *APIP6*, *APIP10*, or *OsAKT1* reduces the PMAP-induced ROS production. In contrast, silencing *APIP5* leads to ROS accumulation.

Another best-characterized ROS related signaling pathway in rice is the Piz-t mediated disease resistance. The R protein Piz-t recognizes the cognate avirulence factor, AvrPiz-t, which is a secreted 108 amino acids polypeptide, by indirect interaction [97,98]. It is known that AvrPiz-t functions to suppress flg22- and chitin-induced ROS generation, and also enhances the susceptibility of rice plants to *M. oryzae* [99]. Recent studies have shown that the AvrPiz-t targets proteins in rice, including APIP5, APIP6, APIP10, APIP12, and OsAKT1, to modulate the host defense responses (Figure 5) [99,100,101,102,103]. Both the APIP6 and APIP10 are RING E3 ubiquitin ligase, which ubiquitinate AvrPiz-t during infection [99,100]. Conversely, AvrPiz-t suppresses the ubiquitin ligase activities of the APIP6 and APIP10. Silencing *APIP6* or *APIP10* reduces flg22-induced ROS production, and enhances the susceptibility of rice plants to *M. oryzae* [99,100]. APIP5, which is a bZIP-type transcriptional factor, interacts with both AvrPiz-t and Piz-t. AvrPiz-t attenuates the transcriptional activity and protein accumulation of the APIP5, while Piz-t stabilizes the APIP5 to prevent necrosis during the necrotrophic stage. Silencing *APIP5* leads to ROS accumulation, cell death, and enhanced resistance to rice blast [101]. AvrPiz-t also interacts with OsAKT1, which is a rice plasma-membrane-localized K^+^ channel protein, and suppresses the OsAKT1-mediated K^+^ currents [103]. OsAKT1, with the cytoplasmic kinase, OsCIPK23, plays a positive role in K^+^ absorption, chitin-induced ROS accumulation, and resistance against *M. oryzae* [103]. It has become apparent that the accumulation of ROS is clearly important in AvrPiz-t triggered immunity. However, at the present time, very little is actually known about how AvrPiz-t suppresses ROS production or how AvrPiz-t interacted proteins regulate ROS accumulation. One possibility has been mentioned that the *OsMT2b* is downregulated in *APIP5* RNAi transgenic plants [70,101]. It will be important to clarify the relationship between AvrPiz-t and its interacted proteins and the ROS generating components or ROS scavengers during immunity in rice plants.

In addition, there are a few of other genes, such as calcium-dependent protein kinase, OsCPK10, DICER-like (DCL) ribonuclease, as well as AGC kinase, OsOxi1, and its interacted protein, OsPti1a, which have been demonstrated to regulate ROS accumulation in the resistance of rice against *M. oryzae* [104,105,106,107]. However, the mechanisms that these genes regulate ROS accumulation remain largely unknown at this time. The most recent report has shown that the rice miRNA, miR398b, could target *Cu/Zn-Superoxidase Dismutasel* (*CSD1*), *CSD2*, and *SODX* to boost H_2_O_2_ accumulation and enhance resistance to *M. oryzae* [108]. These reports suggest that rice plants could regulate ROS accumulation by multiple ways to contribute to resistance against *M. oryzae*.

## 4. Conclusions

It is known that *M. oryzae* needs an intricate ROS accumulation during the appressorium formation and penetration, as well as the neutralization of host-derived ROS during in planta growth. However, there are still many gaps in our knowledge of ROS regulations in *M. oryzae*. Most importantly, how *M. oryzae* suppresses the host-derived ROS accumulation remains largely unclear. Also, whether ROS are related to the biotrophy-necrotrophy switch of rice blast has not yet been confirmed.

On the other hand, there is no doubt that ROS production plays an important role in rice against *M. oryzae*. However, little is known about where the ROS originates from during rice blast. As mentioned above, three enzymes (NADPH oxidase OsRbohB, metallothionein OsMT2b, and NADP-malic enzyme 2 Os-NADP-ME2) are highly regulated and are responsible for ROS accumulation during rice blast infection. However, with the exception of those enzymes, the functions of other ROS generating or scavenging enzymes during rice immunity remain unclear. In addition, barely anything is currently known about the spatio-temporal generation and accumulation of ROS in the rice cells during rice–*M. oryzae* interaction. The detection or measurement of ROS in local and systemic tissues of rice is one of the bottlenecks for analyzing the role of ROS during rice blast. Luminol-chemiluminescence assay by using rice protoplast and DAB (3,3’-Diaminobenzidine) staining are the most common methods to determine ROS in rice. However, it is difficult to use these methods to track ROS generation and accumulation during rice–*M. oryzae* interaction. It might be helpful to use reporters, such as the luciferase reporter, roGFP, or the Hyper sensor, for living cell imaging to answer this important question [19,47,109]. Another important question is why the ROS burst actually occurs. It seems that ROS produced by rice are not a sufficient toxic line of defense to *M. oryzae* even in avirulent interactions or transgenic rice plants with spontaneous lesions [19,85]. It is possible that ROS play a dominant role in signaling, rather than in ROS toxicity. If this is true, it would be important to determine the downstream events of ROS signaling during rice–*M. oryzae* interaction. Moreover, it will be interesting to address the mechanisms that determine the ROS signal specificity in PTI and ETI.
